# 2018 ISCB accomplishments by a senior scientist award

**DOI:** 10.1371/journal.pcbi.1006138

**Published:** 2018-05-17

**Authors:** Christiana N. Fogg, Diane E. Kovats, Ron Shamir

**Affiliations:** 1 Freelance Writer, Kensington, Maryland, United States of America; 2 International Society for Computational Biology; 3 Blavatnik School of Computer Science, Tel Aviv University, Tel Aviv, Israel

**Figure:**
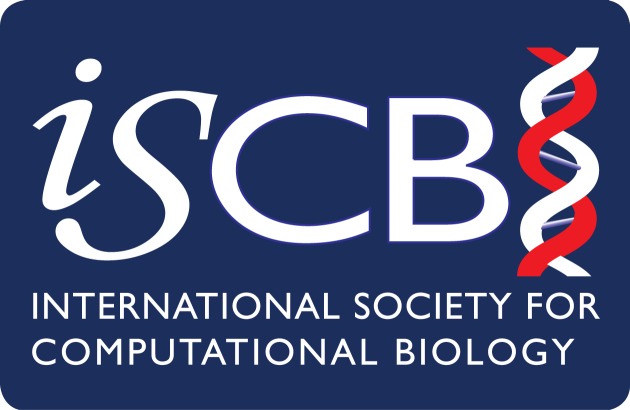


Each year, the International Society for Computational Biology (ISCB) recognizes a leader in the fields computational biology and bioinformatics with the Accomplishments by a Senior Scientist Award. This is the highest award bestowed by ISCB in recognition of a scientist’s significant research, education, and service contributions. Ruth Nussinov, Senior Principal Scientist and Principal Investigator at the National Cancer Institute, National Institutes of Health and Professor Emeritus in the Department of Human Molecular Genetics & Biochemistry, School of Medicine at Tel Aviv University, Israel, is being honored as the 2018 winner of the Accomplishment by a Senior Scientist Award. She will receive her award and present a keynote address at ISCB’s premiere annual meeting, the 2018 Intelligent Systems for Molecular Biology (ISMB) conference in Chicago, Illinois, being held on July 6–10, 2018.

## Ruth Nussinov: In search of biological significance

**Fig 1:**
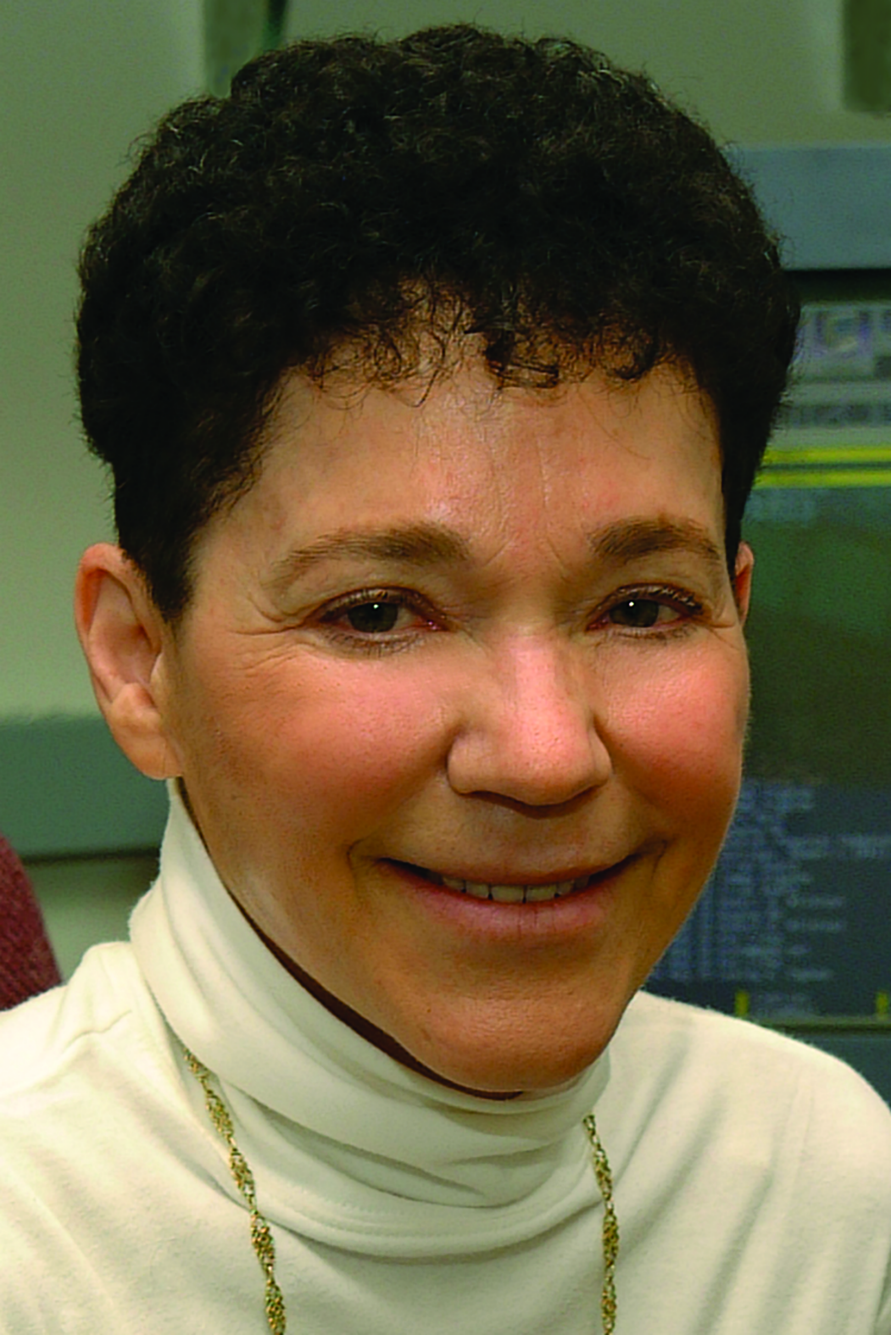
Prof. Ruth Nussinov: Cancer and Inflammation Program, Leidos Biomedical Research, Inc., Frederick National Laboratory for Cancer Research, National Cancer Institute at Frederick, Frederick, Maryland, United States of America. Sackler Institute of Molecular Medicine, Department of Human Genetics and Molecular Medicine, Sackler School of Medicine, Tel Aviv University, Tel Aviv, Israel.

Ruth Nussinov (Fig 1) is a computational biologist with research interests that have touched every aspect of the field, from her PhD research on RNA secondary structure prediction to her visionary work on DNA sequence analysis, to proposing that all protein (and other biomacromolecules) conformations preexist and that all dynamic proteins are allosteric, to her current studies focused on Ras signaling in cancer. Nussinov’s deep intellectual curiosity has guided her research interests throughout her career.

Nussinov was raised in Rehovot, Israel, and attributes her early interest in science to watching her father conduct pioneering agricultural research that focused on adapting crops to the Israeli climate [1,2]. Nussinov’s father, Shmuel Hurwitz, was born in Minsk, Russia, and studied chemistry at Moscow University but later immigrated to Palestine (present-day Israel) after his arrest for Zionist activities. It was here he discovered the great need for agricultural research. He pursued these studies at Berlin University but left Nazi Germany after his graduation in 1933 to found the Agricultural Research Station in Rehovot. Hurwitz was a founding member of the Faculty of Agriculture at the Hebrew University and was recognized for his significant contributions to advancing Israel agriculture with the 1957 Israeli Prize. As a child, Nussinov often joined her father on trips to his field sites, and his devotion to research and intense work ethic influenced her deeply and shaped how she approaches her work.

Nussinov also attributes her success as a scientist to the unwavering support from her husband, Shmuel Nussinov. They married just after she completed her service in the Israeli Army, during which time he was pursuing his graduate studies in particle physics at the Weizmann Institute. Her husband’s research advisor moved to the University of Washington, so Nussinov continued her undergraduate studies there (in microbiology) and went on to pursue her master’s degree in biochemistry at Rutgers University while her husband pursued postdoctoral research at Princeton University. They returned to Israel when Shmuel Nussinov joined the faculty at Tel Aviv University. When they came back to the United States several years later for his sabbatical, Ruth Nussinov enrolled in a PhD program in biochemistry at Rutgers and was mentored by a newly arrived assistant professor named George Pieczenik who had just come from Cambridge (United Kingdom). Nussinov recalled, “He said, ‘You know Ruth, Fred Sanger has just developed a DNA sequencing method and consequently there will be RNA sequences, and we will need an algorithm for the prediction of the secondary structure of RNA.’” She ran with this idea and worked tirelessly to develop the foundational Nussinov dynamic programming algorithm that is still in use today [3]. Nussinov’s PhD research has driven her career-long search for questions that tackle issues of biological significance. She worked relatively independently on her project and was able to graduate in two years, and this early autonomy was critical to shaping her career path as an independent researcher.

Nussinov and her family returned to Israel, and she pursued postdoctoral studies in the Structural Chemistry Department of the Weizmann Institute and made several seminal contributions to DNA sequence analysis. She also worked as a visiting scientist in the Chemistry Department at the University of California, Berkeley, and in the Biochemistry Department at Harvard University. In spite of her impressive body of work and concept-driven approach to scientific inquiry, Nussinov faced difficulties in securing a position at Tel Aviv University in the mid-1980s due to her husband’s existing position at the university and her unconventional, independent career path [4]. In 1985, Nussinov was finally appointed as an associate professor at Tel Aviv University and also became affiliated with the National Cancer Institute (NCI)/National Institutes of Health (NIH). During these early years, she credits her husband for giving her valuable advice about handling criticism from manuscript reviewers. He urged her to trust in her work and to reflect on and revise her manuscripts and resubmit them, as publications matter to the progress of a junior and unknown scientist [2].

One of Nussinov’s most profound contributions to the field is the “conformational selection and population shift” model of molecular recognition [5–9]. She and her colleagues first proposed this model in 1999 as an alternative paradigm to the “induced fit” model of protein–protein interactions. The induced-fit model hypothesizes that conformational changes to a protein occur in a stepwise fashion upon binding to a ligand. In contrast, the conformational selection model portends that unbound molecules exist in all possible structural conformations, but some unbound higher-energy conformations preferentially associate with a binding partner and cause a shift in equilibrium that favors this conformation. This model can explain numerous interactions observed for protein–ligand, RNA–ligand, protein–protein, protein–DNA, and protein–RNA interactions and can explain mechanisms of biological regulation, including oncogenic signaling.

Nussinov is currently focused on the Ras protein and its interactions with effectors, with a particular interest in KRAS-driven adenocarcinomas. She observed that self-association of GTP-dependent K-Ras dimers at different interfaces regulates which effectors bind to the dimers, which can alter downstream activity [10]. Nussinov and her team have also described the critical role of calmodulin selectively binding to the GTP-bound K-Ras4B oncogenic isoform, which promotes the initiation and progression of adenocarcinomas due to full activation of phosphoinositide 3-kinase (PI3Kα)/Akt signaling in addition to the mitogen-activated protein kinase (MAPK) pathway. These mechanistic insights are critical to developing better cancer drugs, and this work was recognized in the “Best of the AACR Journals Collection 2015.” Nussinov is also starting to explore interactions between the human proteome and pathogens, given the growing appreciation of the microbiome on human health.

Nussinov’s impact to the fields of computational biology and bioinformatics is notable. She has published more than 500 articles and has been ranked as a Highly Cited Researcher (ranking among the top 3,000 researchers or 1% across all fields according to Thomson Reuters Essential Science Indicators, http://highlycited.com/ December 2015) with more than 43,000 citations to date. Nussinov has also given over 300 invited talks and continues to maintain an active speaker schedule.

Nussinov serves as the Editor-in-Chief of *PLOS Computational Biology*, and she has also served as an editor and reviewer for numerous leading journals. Her scientific contributions have been recognized through her election as a Fellow of the Biophysical Society (2011) and an ISCB Fellow (2013). Nussinov has been a devoted mentor and advisor to graduate students and trainees throughout her career, and she has mentored dozens of PhD students, including numerous women. She has tried to model her mentorship to how she was trained, and she said, “I very much encourage independence and like for students to suggest a problem to study”.

Nussinov has always felt a close connection with ISCB, and her recognition with the 2018 ISCB Accomplishments by a Senior Scientist Award is a fitting tribute to her contributions to ISCB and to computational biology in general. She said, “I feel that’s where I belong and that’s where I want to be. I care very much about the development and sustainability and contribution of computational biology to all biological, chemical, and physical sciences”.
